# Visualized clinical–radiomics model for predicting the efficacy of surufatinib in hepatic metastases of neuroendocrine neoplasms

**DOI:** 10.3389/fonc.2025.1628054

**Published:** 2025-08-29

**Authors:** Miaomiao Feng, Man Zhao, Xiaoling Duan, Jiaojiao Hou, Qi Wang, Fei Yin

**Affiliations:** ^1^ Department of Gastroenterology, Fourth Hospital of Hebei Medical University, Shijiazhuang, Hebei, China; ^2^ Department of CT and MRI, Fourth Hospital of Hebei Medical University, Shijiazhuang, Hebei, China

**Keywords:** hepatic metastatic neuroendocrine neoplasms, surufatinib, clinical-radiomics model, arterial phase computed tomography, efficacy

## Abstract

**Background:**

Hepatic metastatic neuroendocrine neoplasms (HM-NENs) have few treatment biomarkers and low survival rates. We created a clinical–radiomics fusion model to predict surufatinib efficacy in HM-NENs and presented it as a nomogram, meeting unmet requirements in precision hepatology.

**Methods:**

This study included 76 HM-NEN patients (131 hepatic metastases) treated with surufatinib. SlicerRadiomics was used to extract radiomics features from arterial phase computed tomography (APCT). The least absolute shrinkage and selection operator (LASSO) was used to select radiomics features and calculate a radiomics score (Radscore). Multivariable logistic regression analysis was utilized to create the clinical–radiomics fusion model, which included clinical characteristics and Radscore and was displayed as a nomogram. The area under the receiver operating characteristic curve (ROC) was used to assess model performance, and internal validation was done using the bootstrap resampling approach.

**Results:**

After multivariate logistic regression analysis, the Radscore, Ki67 antigen (Ki67), number of hepatic metastases, and extrahepatic metastasis were included as predictors in the final model. The area under the curve (AUC) of the clinical–radiomics fusion model to predict the response of surufatinib of HM-NENs was 0.928 (95% CI: 0.885 - 0.971). The AUC verified by bootstrap is 0.928 (95% CI: 0.881–0.965), indicating a good performance of the fusion model.

**Conclusion:**

The clinical–radiomics fusion model can effectively identify patients with HM-NENs sensitive to surufatinib therapy. The nomogram provided clinicians with a convenient and dependable tool for decision-making.

## 1 Introduction

Neuroendocrine neoplasms (NENs) are a varied group of cancers that originate from the diffuse neuroendocrine systems (1). NENs can occur anywhere in the body, with lung and gastrointestinal pancreatic NENs being the most common (2, 3). The diagnostic capabilities for NENs have improved over time due to advancements in endoscopic techniques and biomarker technologies. The incidence rate has increased sixfold in the last 40 years (4). At present, the primary treatment modalities for NENs encompass surgical resection, chemotherapy, radiotherapy, and immunotherapy. Beyond these, targeted therapy also serves as a therapeutic option. NENs, characterized by abundant vascular supply, dense capillary networks, and elevated vascular endothelial growth factor (VEGF) expression, present opportunities for anti-angiogenic therapy (5).

Surufatinib, an oral tyrosine kinase inhibitor (TKI) developed in China, targets the VEGF receptor (VEGFR), fibroblast growth factor receptor 1 (FGFR1), and colony-stimulating factor 1 receptor (CSF1R), achieving synergistic antitumor activity through both anti-angiogenic and immunomodulatory effects (6). Surufatinib was approved in China in December 2020 and June 2021 for extra-pancreatic and pancreatic NENs, respectively, based on the SANEN-ep and SANEN-p studies (7, 8). Consequently, surufatinib became China’s first novel NEN-targeted medication and the first TKI worldwide to treat all NEN subtypes. Previous research has shown that NENs have a relative tendency for the liver, regardless of initial location, with approximately 82% of NEN patients developing hepatic metastases during the course of their illness (9, 10). Patients with HM-NENs experienced a 6.1-fold higher risk of mortality than those without metastases, with their 5-year overall survival rate ranging from 13% to 54%. The 5-year survival rate for pancreatic HM-NENs was 13%–54% compared with 75%–99% in the absence of metastasis (11, 12). Therefore, identifying patients who are sensitive to surufatinib is of crucial importance. In this study, a novel radiomics-based approach is proposed to address this need.

How to quickly and non-invasively screen patients who are sensitive to surufatinib is an urgent problem to be solved. APCT was widely utilized for tumor diagnosis and therapy assessment, and it helped identify optimal candidates via pretreatment imaging (13–15). Radiomics, an emerging technology quantifying tumor heterogeneity via imaging features, has shown promise in oncology prognosis (16). Previous studies have shown that radiomics models of liver MRI can help predict the chronicity of drug-induced liver injury (17). This study integrates radiomics features and clinical characteristics to develop a fusion model for predicting the short-term efficacy of surufatinib in HM-NENS, providing clinicians with an accurate, practical, and visible prediction tool.

## 2 Materials and methods

### 2.1 Patients

This retrospective study was approved by the Research Ethical Committee of The Fourth Hospital of Hebei Medical University. We evaluated the medical data of HM-NEN patients treated with surufatinib at the Fourth Hospital of Hebei Medical University from January 2018 to December 2024.

Patients were included if (1) age ≥18 years with histopathologically confirmed NENs, (2) received surufatinib therapy, (3) hepatic metastases with ≥1 measurable lesion at baseline, (4) expected survival ≥3 months, and (5) availability of APCT within 1 month before treatment.

**Figure 1:**
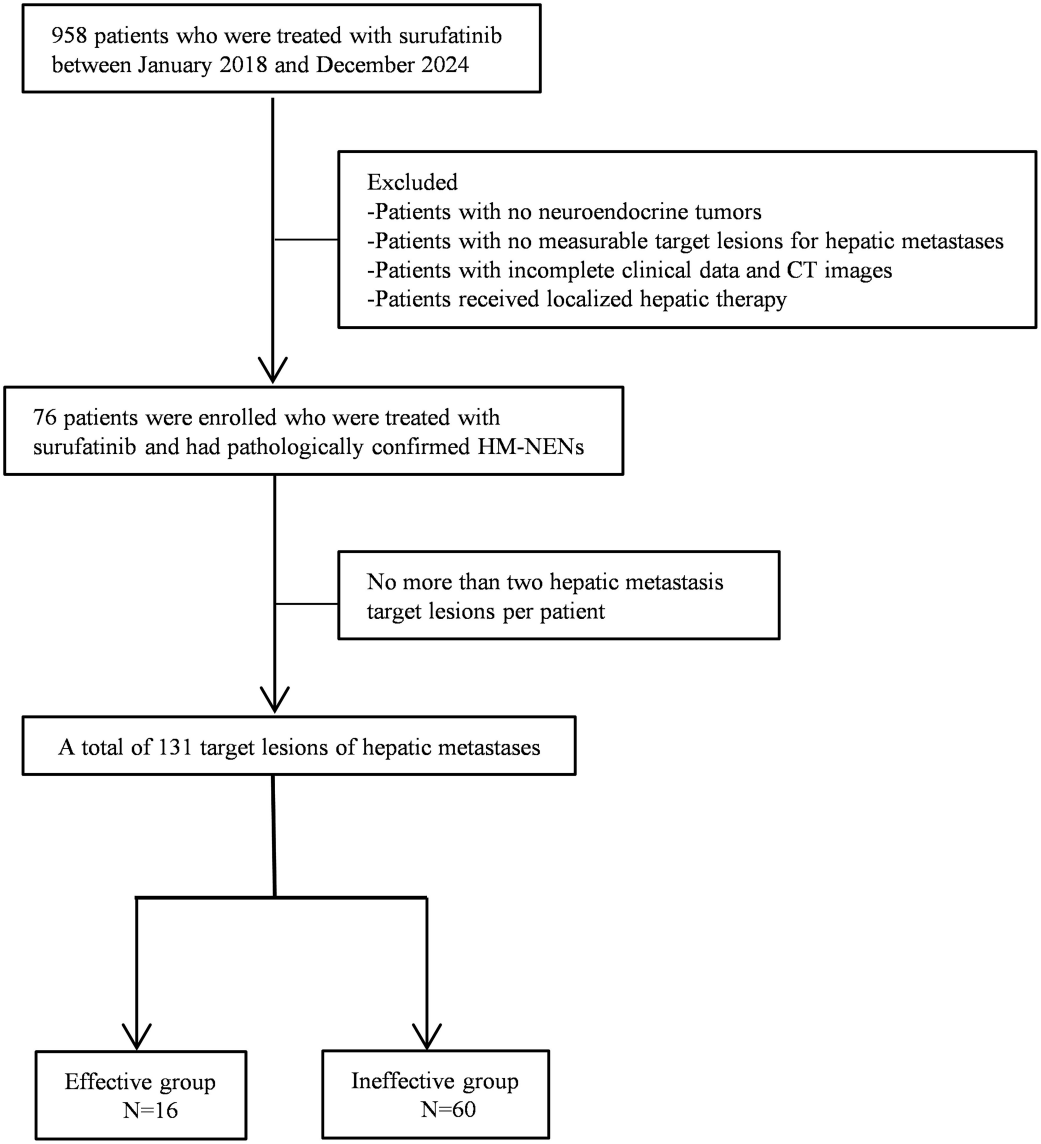
Flowchart of this study.

Patients were excluded if (1) local therapies were administered during follow-up, (2) concurrent malignancies other than NENs, and (3) poor image quality or incomplete clinical data. The patient selection process was shown in **Figure 1**. We included a total of 131 hepatic metastatic lesions in 76 patients with HM-NENs. Of these 76 patients, 16 were effective and 60 were ineffective for treatment with surufatinib.

We conducted a clinical follow-up 3 months after administering surufatinib. mRECIST was a modified response criteria for solid tumors. It was especially well suited to assessing targeted treatment for hepatic metastases by concentrating on changes in arterial phase imaging (18). In this study, we used the mRECIST assessment criteria to determine the efficacy of target lesions of hepatic metastases in patients. The following categories were included: (1) complete response (CR): absence of all target lesions, (2) partial response (PR): at least 30% reduction in the total diameters of target lesions, using the baseline sum diameters as a reference, (3) stable disease (SD): there is not enough shrinking to qualify for PR or enough rise to qualify for PD, and (4) progressive disease (PD): At least 20% rise in the total of the diameters of target lesions, based on the smallest sum on examination, or the appearance of one or more additional lesions. Patients’ responses to surufatinib were divided into two groups: effective (CR + PR) and ineffective (SD + PD).

### 2.2 Clinical characteristics

Demographic characteristics, Ki67, grade, neuron-specific enolase (NSE), gamma-glutamyl transferase (GGT), alpha-fetoprotein (AFP), the diameter of hepatic metastasis, therapy method, surgery, number of hepatic metastases, extrahepatic metastasis, and tumor location were analyzed.

### 2.3 Image segmentation

APCT imaging was conducted using the scan (IQon Spectral CT, Philips Healthcare, Amsterdam, Netherlands), a 256-slice CT system, with the following parameters: tube voltage of 120 kVp, automatic tube current modulation, slice thickness of 5.0 mm, reconstruction increment of 5.0 mm, gantry rotation time of 0.5 s, pitch factor of 0.973, and a standard reconstruction algorithm with a reconstruction section thickness of 1 mm. The APCT images were imported into the 3D Slicer 5.6.2 software (https://www.slicer.org/) in DICOM format. All images were resampled to the same size of 1 × 1 × 1 mm³ and denoised using wavelet filtering. The region of interest (ROI) was manually designated by two clinicians with over 10 years of clinical experience, who were uninformed of the clinicopathological variables and prognosis of the patients. When differences of opinion arose during the drawing process, these were resolved via mutual negotiation. The largest diameter of the hepatic metastases (>1 cm measurable lesion) was selected, and the volume of interest (VOI) was manually drawn layer by layer along the tumor’s edge. A maximum of two hepatic metastases were chosen as target lesions per patient. To evaluate the intra- and inter-observer repeatability of ROI segmentation, 46 randomly selected hepatic metastases were re-segmented by the same observer (observer 1) twice at 2-week intervals. Simultaneously, the 46 randomly selected hepatic metastases were segmented by another observer (observer 2) to evaluate inter-observer repeatability.

### 2.4 Feature extraction and model construction

3D image texture features were extracted from 131 hepatic metastases using the SlicerRadiomics plugin (https://github.com/AIM-Harvard/SlicerRadiomics) in 3D Slicer 5.6.2. The Z-score method was used to standardize the radiomics features. We used intra- and inter-group correlation coefficients (ICC) to evaluate feature extraction repeatability between observers. Radiomic features with ICC <0.75 were excluded. Spearman correlation analysis was used to remove strongly correlated radiomics features, removing those with correlations larger than 0.90. Tenfold cross-validation was performed to determine the hyperparameters in LASSO (19). Finally, a Radscore was calculated by linearly combining the selected features. For the clinical characteristics, multivariate analysis using a backward stepwise selection method was executed via logistic regression to identify independent predictors of efficacy with *p <*0.05. The Radscore was integrated with clinical characteristics to construct a fusion model. We calculated the AUC, sensitivity, and specificity of the model to evaluate the performance of the model. Internal validation of the model was conducted using bootstrap with 1,000 iterations. The bootstrap approach is crucial in our small-sample studies as it does not need to rely on large-sample assumptions and generates pseudo-samples through resampling to reconstruct the statistical distributions, thus enabling a robust estimation of uncertainty (20). The mean values obtained from these 1,000 resampling procedures were used to evaluate the model’s performance. It ensured a complete evaluation of the model’s generalization capacity as well as a realistic estimation of its discriminatory power over multiple data subsets typical of the underlying population.

### 2.5 Statistical analyses

The statistical analysis was conducted using R 4.4.2 (https://cran.r-project.org/). Continuous data with a normal distribution were reported as mean ± standard deviation (SD). Continuous data that did not follow a normal distribution were reported as median (upper and lower quartiles). Categorical data were represented using frequencies and percentages. Chi-square test was used to compare categorical variables. Mann–Whitney *U*-test was used to compare continuous variables that did not conform to a normal distribution. For variables with *p <*0.05 in the univariate analysis, we performed a multivariate logistic regression using backward stepwise selection based on the Wald statistic. Correlation analysis was conducted using Spearman correlation. Calibration curves were plotted to assess the fit of the model. Decision curve analysis (DCA) was used to quantify the NEN benefit under different threshold probabilities and to assess the clinical utility of the model. A *p*-value <0.05 was considered statistically significant.

## 3 Results

### 3.1 Patients’ characteristics

A total of 76 patients with 126 hepatic metastases were eligible for inclusion in this study. The mean age was 62 years, and 48.68% of the patients were male. According to mRECIST, the therapeutic effect of surufatinib was CR/PR in 16 (21.05%) patients and SD/PD in 60 (78.95%) patients. The patients’ characteristics are shown in **Table 1**.

**Table 1:** Patients’ characteristics.

Variable	(Total) *N* = 76
Age^a^	61.88 ± 11.15
Sex	
Male	37 (48.68)
Female	39 (51.32)
NSE	
≤100	15 (19.74)
>100	61 (80.26)
Ki67 [median (IQR)]	27.50 [7.50, 80.00]
AFP, ng/mL [median (IQR)]	4.60 [2.72, 10.20]
GGT, U/L [median (IQR)]	28.75 [18.45, 53.22]
Grade	
1	7 (9.21)
2	29 (38.16)
3	8 (10.53)
4	32 (42.11)
Number of hepatic metastases	
Oligometastasis	29 (38.16)
Multiple metastases	36 (47.37)
Diffuse metastasis	11 (14.47)
Diameter of the hepatic metastasis	
1–3 cm	52 (68.42)
3–5 cm	21 (27.63)
>5 cm	3 (3.95)
Tumor location	
EP-NEN	47 (61.84)
PNEN	29 (38.16)
Extrahepatic metastasis	
No	43 (56.58)
Yes	33 (43.42)
Surgery	
No	52 (68.42)
Yes	24 (31.58)
Therapy method	
Surufatinib + octreotide + chemotherapy and/or immunotherapy	8 (10.53)
Surufatinib + octreotide	18 (23.68)
Surufatinib + chemotherapy and/or immunotherapy	36 (47.37)
Surufatinib	14 (18.42)
Response	
CR + PR	16 (21.05)
SD + PD	60 (78.95)

Unless otherwise indicated, the data are numbers of patients, with percentage in parentheses.

NSE, neuron-specific enolase; Ki67, Ki67 antigen; AFP, alpha-fetoprotein; GGT, gamma-glutamyl transferase; EP-NEN, extra-pancreatic neuroendocrine neoplasms; P-NEN, pancreatic neuroendocrine neoplasms.

a Data are means ± standard deviation.

In the 76 patients eligible for this study, there were 131 target hepatic metastases. There were no significant differences in AFP, GGT, and surgery between the effective group (CR + PR) and the ineffective group (SD + PD). The other baseline characteristics, including the diameter of the hepatic metastasis, number of hepatic metastases, extrahepatic metastasis, tumor location, NSE, Ki67, grade, and Radscore, were substantially different between the two groups (**Table 2**).

**Table 2:** Baseline characteristics of effective and ineffective groups in the HM-NENs.

Variable	Effective group (*n* = 27)	Ineffective group (*n* = 104)	*p*
NSE			0.010
≤100	10 (37.04)	14 (13.46)	
>100	17 (62.96)	90 (86.54)	
Ki67 [median (IQR)]	15.00 [5.00, 30.00]	30.00 [7.25, 80.00]	0.050
Grade			0.010
1	3 (11.11)	10 (9.62)	
2	16 (59.26)	36 (34.62)	
3	4 (14.81)	9 (8.65)	
4	4 (14.81)	49 (47.12)	
Tumor location			0.035
EP-NEN	11 (40.74)	68 (65.38)	
PNEN	16 (59.26)	36 (34.62)	
Diameter of the hepatic metastasis			0.016
1–3 cm	24 (88.89)	62 (59.62)	
3–5 cm	3 (11.11)	35 (33.65)	
>5 cm	0 (0.00)	7 (6.73)	
Number of hepatic metastases			0.018
Oligometastasis	12 (44.44)	35 (33.65)	
Multiple metastases	15 (55.56)	48 (46.15)	
Diffuse metastasis	0 (0.00)	21 (20.19)	
Extrahepatic metastasis			0.002
No	23 (85.19)	52 (50.00)	
Yes	4 (14.81)	52 (50.00)	
Surgery			0.531
No	20 (74.07)	68 (65.38)	
Yes	7 (25.93)	36 (34.62)	
AFP, ng/mL[median (IQR)]	3.88 [2.82, 5.90]	5.32 [2.72, 12.07]	0.182
GGT, U/L[median (IQR)]	24.30 [18.90, 44.65]	33.95 [19.10, 67.97]	0.152
Radscore [median (IQR)]	0.78 [0.38, 1.15]	1.81 [1.25, 2.38]	<0.001

Unless otherwise indicated, the data are numbers of patients, with percentage in parentheses.

HM-NENs, hepatic metastatic neuroendocrine neoplasms; NSE, neuron-specific enolase; Ki67, Ki67 antigen; AFP, alpha-fetoprotein; GGT, gamma-glutamyl transferase; EP-NEN, extra-pancreatic neuroendocrine neoplasms; P-NEN, pancreatic neuroendocrine neoplasms.

### 3.2 Feature extraction and radiomics signature building

A total of 851 radiomics features were extracted for each lesion. Among them, there were 14 shape features, 18 first-order features, 24 gray-level co-occurrence matrix (GLCM) features, 16 gray-level size zone matrix (GLSZM) features, 16 gray-level run length matrix (GLRLM) features, five neighboring gray tone difference matrix (NGTDM) features, 14 gray-level dependence matrix (GLDM) features, and 744 wavelet features. Features with ICC of less than 0.75 were excluded, and Spearman correlation analysis was used to remove duplicate features with correlation coefficients larger than 0.90 with other features. After analysis, 60 radiomics features were selected, as shown in **Supplementary Table S1**. The best radiomics features closely related to efficacy were selected by using LASSO with 10-fold cross-validation. Original_RootMeanSquared, Original_Correlation, Original_LongRunLowGrayLevelEmphasis,Original_Busyness,Wavelet_LLH_Median,Wavelet_LLH_ClusterShade,Wavelet_LLH_Imc1,WaveletH_LLHH_RunVariance, and Wavelet_LLH_SizeZoneNonUniformityNormalized were the optimal radiomics features. **Supplementary Figure S1** shows the procedure of LASSO screening. The calculation formula of Radscore is shown below.


Radscore=−0.429×Original_RootMeanSquared+1.054×Original_Correlation+0.023×Original_LongRunLowGrayLevelEmphasis+0.406×Original_Busyness−0.162×Wavelet_LLH_Median+0.118×Wavelet_LLH_ClusterShade+0.960×Wavelet_LLH_Imc1−0.948×WaveletH_LLHH_RunVariance+0.308×Wavelet_LLH_SizeZoneNonUniformityNormalized

**Figure 2:**
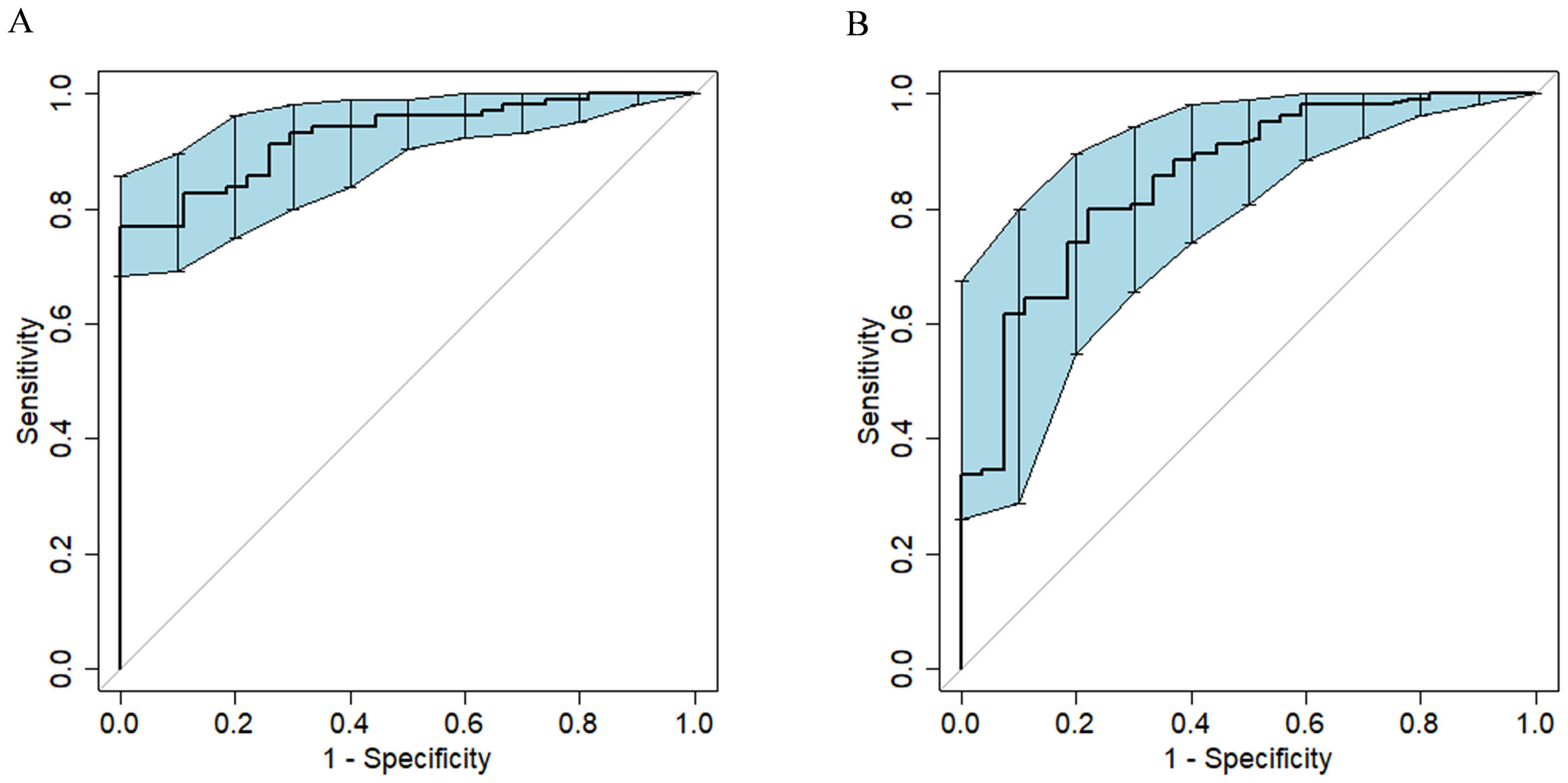
ROC curves of the model for predicting the efficacy of surufatinib in the treatment of HM-NENs. **(A)** Clinical–radiomics fusion model. **(B)** Radiomics model.

Radscore was significantly associated with the response of HM-NENs to surufatinib (*p* = 0.000), with an AUC of 0.852 (95% confidence interval [CI],0.767–0.922) (**Figure 2B**).

### 3.3 Development of visualized predictive models

**Figure 3:**
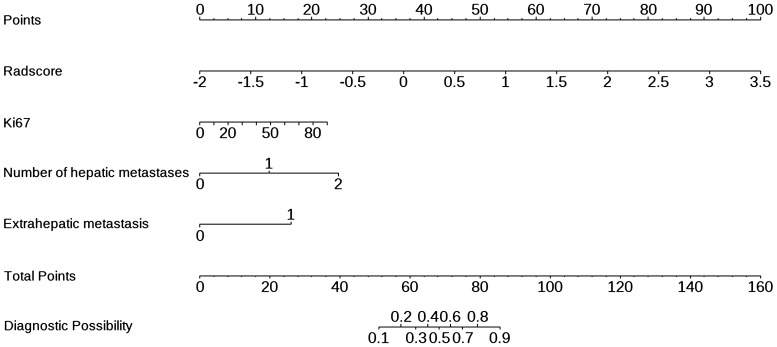
Nomogram for predicting the efficacy of surufatinib in HM-NENs.

**Figure 4:**
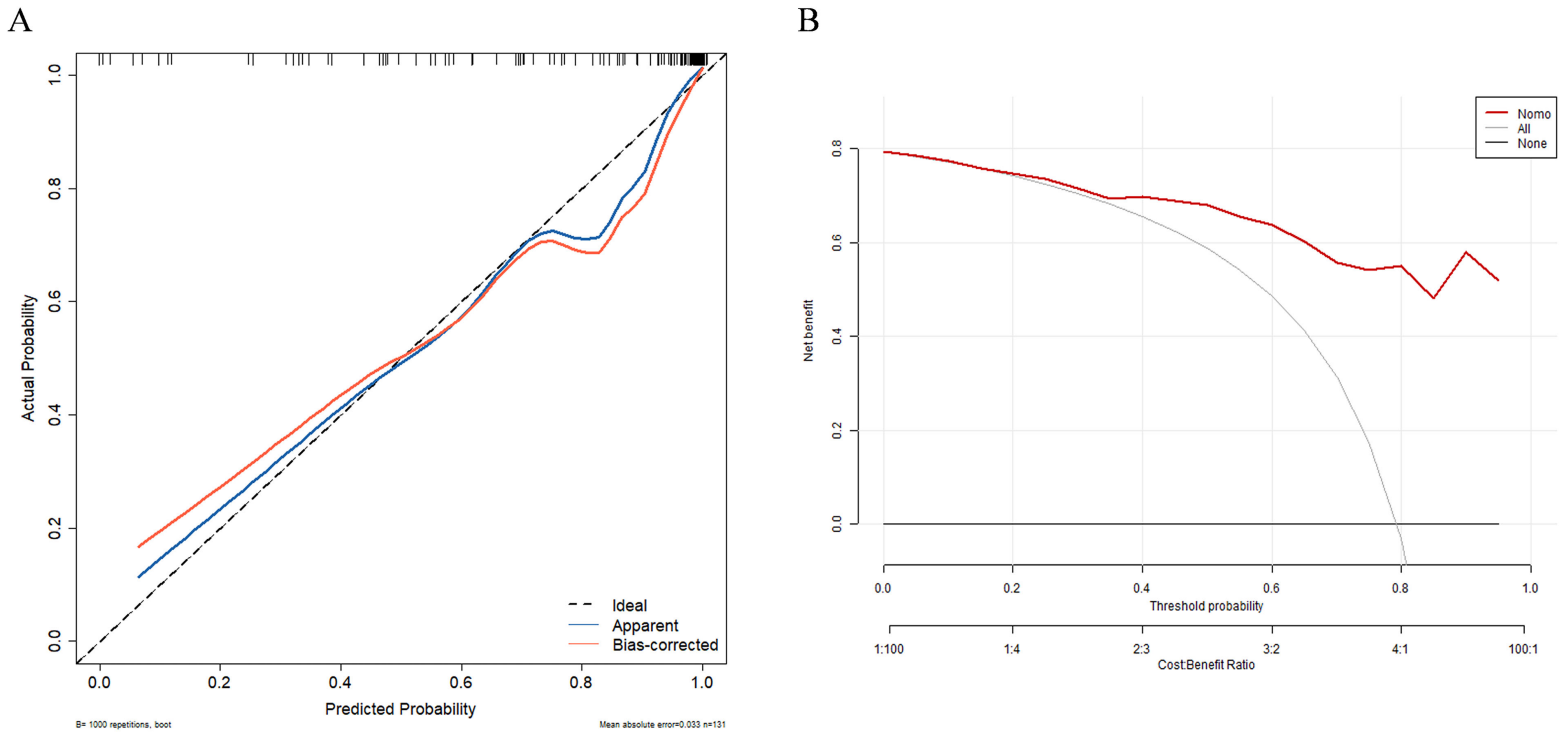
Calibration curve **(A)** and decision curve **(B)** of the clinical–radiomics fusion model. Calibration curves indicate the goodness-of-fit of the fusion model.

In univariate and multivariate logistic regression analysis (**Table 3**), Radscore (*p* = 0.000), Ki67 (*p* = 0.005), the number of hepatic metastases (*p* = 0.050), and extrahepatic metastasis (*p* = 0.009) were identified as independent clinical risk variables. A fusion model that incorporated the independent predictors was developed. The fusion model was displayed as a nomogram, providing clinicians with an effective tool for identifying HM-NEN patients who were susceptible to surufatinib treatment (**Figure 3**). The AUC of the fusion model was 0.928 (95% CI: 0.885–0.971). The AUC verified by bootstrap is 0.928 (95% CI: 0.881–0.965) (**Figure 2**). **Figure 4A** shows the calibration curve of the fusion model. The ideal curve aligned with the calibration prediction curve, validating the superior goodness-of-fit of the fusion model. The decision curve of analysis (DCA) is shown in **Figure 4B**, where the horizontal axis represents the risk threshold probability, and the vertical direction represents the normalized NEN benefit. DCA indicated that the fusion model provided a greater NEN clinical benefit at the appropriate risk thresholds.

**Table 3:** Univariate and multivariate logistic regression analyses of the risk factors for the efficacy of HM-NENs.

Variable	Univariable analysis		Multivariable analysis	
	OR (95% CI)	*P*-value	OR (95% CI)	*P*-value
Radscore	7.273 (3.431–18.94)	0	8.808 (3.433–30.18)	0.00s0*
Ki67	1.02 (1.006–1.036)	0.008	1.035 (1.012–1.063)	0.005*
Grade	1.712 (1.138–2.667)	0.012		
GGT	1.004 (0.999–1.012)	0.242		
AFP	1.068 (1.009–1.18)	0.114		
Diameter of the hepatic metastasis	4.936 (1.724–21.01)	0.01	4.050 (1.058–22.29)	0.067
Therapy method	1.534 (0.95–2.508)	0.082		
Surgery	1.513 (0.606–4.16)	0.394		
Number of hepatic metastases	2.007 (1.051–4.059)	0.042	3.795 (1.108–16.58)	0.050*
NSE	3.782 (1.425–9.941)	0.007		
Extrahepatic metastasis	5.75 (2.041–20.63)	0.002	10.57 (2.137–79.68)	0.009*
Sex	0.898 (0.378–2.1)	0.804		
Age	1.003 (0.964–1.042)	0.898		
Tumor location	0.364 (0.149–0.858)	0.022		

HM-NENs, hepatic metastatic neuroendocrine neoplasms; NSE, neuron-specific enolase; Ki67, Ki67 antigen; AFP, alpha-fetoprotein; GGT, gamma-glutamyl transferase.

*p < 0.05.

## 4 Discussion

There were individual differences in the responses of patients with HM-NENs to treatment with surufatinib. In our study, more than half of the patients were ineffective on surufatinib (60 out of 76 patients). Therefore, it was necessary to construct an efficient, simple, and visualized prediction tool for clinicians to predict in advance how patients will benefit from the drug and reduce their financial burden. Radiomics, as a novel technology, has the potential to serve as a quantitative decision support tool, and its integration with clinically relevant information could greatly improve clinical prediction capabilities (21, 22). In this study, a clinical–radiomics fusion model was developed and illustrated by a nomogram. The AUC of the fusion model was validated within bootstrap as 0.928 (95% CI: 0.881 - 0.965), demonstrating good predictive performance. Clinicians using this nomogram could efficiently identify patients with HM-NENs who would benefit from surufatinib.

So far, there have been almost no studies on the therapeutic efficacy of surufatinib in HM-NENs. In our study, we developed a personalized visualization combination model that predicted the therapeutic response of hepatic metastases to surufatinib based on the patient’s pre-dose APCT. Our study aligned with Lambin’s “radiomics signature” concept, where multidimensional data integration enabled a more comprehensive assessment of tumor treatment response (23). Poor therapy response was predicted by elevated Radscore and Ki67 as well as more hepatic and extrahepatic metastases.

While tumor diameter was not an independent predictor of surufatinib efficacy in our study, other HM-NEN prognostic studies highlight the value of quantitative tumor load assessments. One study analyzed the value of quantitative tumor load analysis on baseline MRI in predicting survival in patients with HM-NENs receiving intra-arterial therapy and found that tumor load was a strong independent prognostic factor for overall survival (24, 25). Tumor load was determined by a combination of tumor diameter and the number of tumors; however, our research revealed that tumor diameter was not an independent predictor of surufatinib efficacy. This was attributed to the small percentage of patients in our study who had tumor diameters larger than 5 cm, which totaled only three (3.95%). Previous studies have shown that larger hepatic metastases (>5 cm) exhibited hypoxic microenvironments with lower drug penetration, which was associated with worse responses to targeted therapies (26, 27). The number of hepatic metastases as an independent predictor also reflected tumor load in one way. In deeper subsequent studies, as Zhang et al. demonstrated, circLIFR-007 exerts inhibitory effects on the proliferation and metastasis of breast cancer cells both *in vivo* and *in vitro (* 28). As Zeng et al. demonstrated, circMYBL2 promoted the tumorigenesis and aggressiveness of breast cancer (29). Along this line, we can also explore the molecular mechanisms associated with HM-NENs, which may provide a novel targeted therapy for patients with HM-NENs.

Our study does have some limitations: (1) a retrospective single-center design with a small sample size necessitates testing in multi-center studies; single-center data may introduce selection bias, (2) the use of APCT images alone—integration with multiparametric MRI may improve accuracy, and (3) variations in scan parameters, reconstruction algorithms, and segmentation methods pose feature reproducibility challenges. While standardized techniques decreased these impacts, more preprocessing and feature extraction optimization are required. This study solely looked at the radiomics within the tumor and ignored the area surrounding it. In the future, the region of interest (ROI) around the tumor should be investigated to extract more radiomics properties.

Despite these limitations, this study demonstrated notable strengths. In the field of HM-NENs and surufatinib efficacy prediction, research employing clinical–radiomics models and visualized nomograms remained relatively scarce. This study pioneered a novel exploratory pathway and established a foundation for subsequent multicenter, large-sample investigations. Radiomics technology enabled the extraction of radiomics features from CT images, and the models integrating clinical characteristics demonstrated effective predictive capabilities for surufatinib therapeutic response. The clinical–radiomics fusion models exhibited greater objectivity and comprehensiveness than traditional clinical evaluation methods. This advancement not only facilitated more informed clinical decision-making during treatment planning but also offered innovative perspectives and methodologies for personalized therapeutic strategies.

In conclusion, the APCT-based clinical–radiomics model with an AUC of 0.928 offered a noninvasive and visible analytical tool for the customized therapy of HM-NENs. Continued advances in radiomics technology promise to increase its role in precision medicine, eventually increasing treatment outcomes and survival rates.

## Data Availability

The raw data supporting the conclusions of this article will be made available by the authors, without undue reservation.
