# Supplementary material for: Visualized clinical–radiomics model for predicting the efficacy of surufatinib in hepatic metastases of neuroendocrine neoplasms
Source: Front Oncol. 2025 Aug 29;15:1628054. doi: 10.3389/fonc.2025.1628054 (PMC12425703; doi:10.3389/fonc.2025.1628054)
Supplement: Supplementary file 1 [file DataSheet1.zip › Supplementary material.doc]

**TableS 1**Radiomics features selected by ICC and spearman correlation analysis

|  | **name** | **rename** |
| --- | --- | --- |
| 1 | original_LeastAxisLength | V1 |
| 2 | original_MajorAxisLength | V2 |
| 3 | original_SurfaceVolumeRatio | V3 |
| 4 | original_90Percentile | V4 |
| 5 | original_InterquartileRange | V5 |
| 6 | original_Maximum | V6 |
| 7 | original_Minimum | V7 |
| 8 | original_Range | V8 |
| 9 | original_RobustMeanAbsoluteDeviation | V9 |
| 10 | original_RootMeanSquared | V10 |
| 11 | original_ClusterShade | V11 |
| 12 | original_Correlation | V12 |
| 13 | original_DifferenceAverage | V13 |
| 14 | original_Idmn | V14 |
| 15 | original_Idn | V15 |
| 16 | original_Imc1 | V16 |
| 17 | original_InverseVariance | V17 |
| 18 | original_MaximumProbability | V18 |
| 19 | original_SumAverage | V19 |
| 20 | original_DependenceEntropy | V20 |
| 21 | original_DependenceVariance | V21 |
| 22 | original_LargeDependenceHighGrayLevelEmphasis | V22 |
| 23 | original_LongRunLowGrayLevelEmphasis | V23 |
| 24 | original_RunEntropy | V24 |
| 25 | original_RunVariance | V25 |
| 26 | original_GrayLevelNonUniformity_2 | V26 |
| 27 | original_GrayLevelNonUniformityNormalized_1 | V27 |
| 28 | original_LargeAreaLowGrayLevelEmphasis | V28 |
| 29 | original_ZoneEntropy | V29 |
| 30 | original_ZonePercentage | V30 |
| 31 | original_ZoneVariance | V31 |
| 32 | original_Busyness | V32 |
| 33 | original_Contrast_1 | V33 |
| 34 | wavelet_LLH_90Percentile | V34 |
| 35 | wavelet_LLH_Maximum | V35 |
| 36 | wavelet_LLH_Median | V36 |
| 37 | wavelet_LLH_TotalEnergy | V37 |
| 38 | wavelet_LLH_ClusterShade | V38 |
| 39 | wavelet_LLH_Contrast | V39 |
| 40 | wavelet_LLH_Correlation | V40 |
| 41 | wavelet_LLH_Idmn | V41 |
| 42 | wavelet_LLH_Imc1 | V42 |
| 43 | wavelet_LLH_InverseVariance | V43 |
| 44 | wavelet_LLH_JointEnergy | V44 |
| 45 | wavelet_LLH_MCC | V45 |
| 46 | wavelet_LLH_DependenceEntropy | V46 |
| 47 | wavelet_LLH_DependenceNonUniformityNormalized | V47 |
| 48 | wavelet_LLH_DependenceVariance | V48 |
| 49 | wavelet_LLH_LargeDependenceEmphasis | V49 |
| 50 | wavelet_LLH_LargeDependenceHighGrayLevelEmphasis | V50 |
| 51 | wavelet_LLH_LargeDependenceLowGrayLevelEmphasis | V51 |
| 52 | wavelet_LLH_RunVariance | V52 |
| 53 | wavelet_LLH_GrayLevelNonUniformityNormalized_1 | V53 |
| 54 | wavelet_LLH_SizeZoneNonUniformityNormalized | V54 |
| 55 | wavelet_LLH_SmallAreaLowGrayLevelEmphasis | V55 |
| 56 | wavelet_LLH_ZoneVariance | V56 |
| 57 | wavelet_LLH_Busyness | V57 |
| 58 | wavelet_LLH_Coarseness | V58 |
| 59 | wavelet_LLH_Contrast_1 | V59 |
| 60 | wavelet_LLH_Strength | V60 |

**FigureS 1**(A)The optimal parameter [λ] selection in the LASSO model employed tenfold cross-validation using a minimum criteria approach. Dotted vertical lines represent the optimal values of λ.(B)LASSO coefficient profiles of 60 radiomics features. The plot was created using a logarithmic scale for the lambda values. A vertical line was added to indicate the lambda value selected through tenfold cross-validation. This optimal lambda value led to the identification of 9 features with nonzero coefficients
